# Supporting the diagnosis of infantile colic by a point of care measurement of fecal calprotectin

**DOI:** 10.3389/fped.2022.978545

**Published:** 2022-09-29

**Authors:** Henning Sommermeyer, Malgorzata Bernatek, Marcin Pszczola, Hanna Krauss, Jacek Piatek

**Affiliations:** ^1^Department of Health Sciences, Calisia University, Kalisz, Poland; ^2^Department of Genetic and Animal Breeding, Faculty of Veterinary Medicine and Animal Science, Poznan University of Life Sciences, Poznan, Poland

**Keywords:** colicky babies, gut microbiota, cesarean section, fecal calprotectin (FC), gut inflammation, infantile colic, point of care diagnostic, vaginal birth

## Abstract

**Background:**

Infantile colic (IC) is a condition characterized by extensive crying which affects about 20% of all infants during their first months of life. Most pediatricians diagnose IC only based on their clinical experience.

**Aim:**

Investigating if a measurement of fecal calprotectin can support the diagnosis of IC.

**Methods:**

The crying behavior of newborns was assessed using the Wessel's criteria. Fecal calprotectin levels were measured in non-colicky and colicky babies using a standard test that can be used at the time and place of patient care (point of care (PoC) measurement).

**Results:**

Colicky babies were found to have significantly elevated fecal calprotectin levels. Calprotectin levels were not influenced by gender, type of feeding, gestation age or birth weight. However, significantly elevated fecal calprotectin levels were found in cesarean section born babies. Fecal calprotectin ≥100 μg/g correlated with a colicky status of an infant while those <100 μg/g indicated a non-colicky status the error margin was 11.2 and 13.2%, respectively. Combining data of fecal calprotectin with information about the type of delivery made it possible to determine the colicky status in vaginally-born infants with fecal calprotectin ≥100 μg/g with an accuracy of 97.8%. As elevated fecal calprotectin levels in cesarean-born infants can be caused by IC, but also by the disturbed gut microbiota commonly found in these babies, the accuracy of diagnosing the colicky status of a cesarean-born infant with calprotectin levels ≥100 μg/g was less accurate (accuracy rate of 76.5%).

**Conclusion:**

Data from the study suggest that measuring fecal calprotectin should be considered by pediatricians to support the diagnosis of IC.

The study was registered at ClinicalTrials.gov under NCT04666324.

## Introduction

Infants with IC exhibit prolonged duration of inconsolable crying or fussing without a clear identifiable organic cause. It is a benign condition which peaks when the infant is around 6 weeks of age and in most cases resolves by 3–6 months of age (1). IC is causing significant stress for parents and has been identified as a risk-factor for (i) maternal depression (2), (ii) early termination of breastfeeding (3) and (iii) shaken baby syndrome (4). Reported occurrence rates vary widely (3–40%), which might be related to the utilization of different diagnostic criteria employed in the respective studies (5). In 1954, Wessel et al. (6) defined colicky babies as those crying or fussing for more than 3 h a day, for more than 3 days per week, for more than 3 weeks (Wessel's rule of three). Asking parents to wait for 3 weeks until it is possible to establish a diagnosis was found to be impractical, which resulted in a set of modified Wessel criteria, in which the duration of symptoms was reduced to 1 week (7, 8). As Wessel's rule of three and the modified Wessel criteria failed to address the benign character of IC, it was soon felt that it had to be classified as a functional gastrointestinal disorder (FGID) under the Rome diagnostic criteria (Rome III criteria) (9). FGID comprise chronic or recurrent symptoms that occur in the absence of any anatomic abnormality, inflammation or tissue damage. The Rome III criteria for IC applies to newborns from birth to 4 months of age and must include paroxysms of irritability, fussing/crying that starts and stops without any obvious cause, with episodes lasting three or more hours a day, occurring 3 days a week, for at least 1 week and no failure to thrive. However, the Rome III criteria were also identified as not overly useful in clinical practice as the 3 h cut-off value for crying or fussing was arbitrary. Moreover, reporting of the infant's crying by the use of crying diaries was found to be challenging for parents and often biased by the stress level of the reporting parent. In 2017, the Rome IV criteria were published, providing diagnostic criteria for clinical purposes which were complemented by additional criteria for research purposes (10). The Rome IV criteria are “An infant who is < 5 months of age when symptoms start and stop, recurrent and prolonged periods of infant crying, fussing or irritability reported by caregivers that occur without any obvious cause and cannot be prevented or resolved by caregivers; no evidence of infant failure to thrive, fever or illness”. Despite all these developments in the area of IC diagnosis, none of the diagnostic criteria described above have established themselves as a gold standard in clinical practice. In daily life it is impracticable for parents to assess and document crying duration for longer time periods using detailed diaries (5, 11). In a recent survey performed among Turkish pediatricians the vast majority of participants stated that they mostly diagnosed IC based on their clinical experience without the use of strict diagnostic criteria (12).

The etiology of IC is still not fully understood; however, a growing number of studies have identified a dysbiosis of the gut microbiota as a potential cause for the condition (13). Studies comparing the gut microbiota of colicky babies with those of age-matched non-colicky babies identified significant differences (14, 15). In infants with IC, the bacterial colonization of the gut has been found to be retarded, the diversity of bacterial strains to be limited and the stability of the composition to be reduced. That a disturbed gut microbiota might be involved in IC is also supported by studies demonstrating that the supplementation of the gut microbiota by products containing bacterial probiotics is improving the crying behavior of colicky babies (16, 17).

It has been shown that the dysbiosis found in infants with colic is associated with low-grade systemic inflammation characterized by increased serum concentrations of interleukin-8, monocyte chemotactic protein-1 and macrophage inflammatory protein 1β when compared to non-colicky infants (18). A recent meta-analysis came to the conclusion that the efficacy of probiotics in managing IC is related to their anti-inflammatory properties (19).

An established non-serum marker for gut inflammation is fecal calprotectin (20). Calprotectin is a calcium- and zinc-binding protein mainly found within neutrophils. The presence of calprotectin in feces is a consequence of neutrophil migration into the gastrointestinal tissue due to inflammatory processes. Fecal calprotectin concentration demonstrates good correlation with intestinal inflammation and is commonly used as a biomarker for gastrointestinal disorders. It has been found that fecal calprotectin is elevated in colicky babies (21) and that treatment with products containing bacterial probiotics which improves the crying behavior in colicky babies is also lowering fecal calprotectin (22, 23).

The aim of the present study was to evaluate if measuring of fecal calprotectin can contribute to the diagnosis of IC. For this purpose, infants were evaluated for IC by using the Wessel criteria and their level of fecal calprotectin was determined by using a commercially available PoC fecal calprotectin test.

## Materials and methods

### Collection of basic patient data and diagnosis of infantile colic

Recruitment to this study took place at the GP Clinic “Pro Familia”, 62-028 Kozieglowy, Poland and the GP Clinic “Panaceum” 27-230 Brody, Poland between December 2020 and March 2022. Data presented have been acquired as part of a study which was approved by the Ethics Committee for studies involving humans of the Calisia University, Poland (project identification code 2/2020, approved on 20.10.2020) and which have been registered at ClinicalTrials.gov (NCT04666324). Written informed consent was obtained from the infants' parents.

The crying behavior of all newborns (aged 3–6 weeks) was assessed as part of the standard set of examinations. Babies, for whom parents provided consent for participating in the study, were assessed for eligibility to become enrolled into the study. Inclusion criteria for the study were diagnosis as colicky baby based on the Wessel criteria and age 3–6 weeks. Exclusion criteria were previous treatment with probiotics, synbiotics, antibiotics, or crying because of organic causes.

At the enrollment into the study, patient information regarding type of delivery, weight at birth, gestational age, and feeding details were assessed with a questionnaire completed by parents supported by midwives or nutritionists.

### Measurement of fecal calprotectin

Fecal calprotectin level determination was performed using a PoC measurement, which is a method which allows, if necessary or wished, to perform the measurement at the time and place of patients care. Stool samples for determination of fecal calprotectin were taken from all enrolled patients. Collection of samples was performed either during the enrollment physician visit with the cooperation of parents assisted by a nurse, or at home by parents according to instructions provided for sample collection. In all cases samples were immediately frozen at −18°C. Samples were transported by using special cooling containers. Stool samples were stored at −18°C until further processing. Single-use Calex^®^ Caps (Bühlmann Laboratories, Schönenbuch, Switzerland) were used according to the manufacturer's instructions to prepare samples for measurements. Calprotectin concentrations were determined using QB^®^fCAL extended test (Bühlmann Laboratories, Schönenbuch, Switzerland) in combination with a Quantum Blue^®^ Reader II BI-POCTR-ABS (Bühlmann Laboratories, Schönenbuch, Switzerland).

### Statistical analyses

Statistical analyses and data processing were performed in the R environment (24) using the tidyverse package (25). Basic statistics for comparing significance of the differences between non-colicky and colicky patients were performed using *t*-test and chi-square tests implemented in the rstatix package (26).

#### Relationship between calprotectin level and colicky status

The impact of the response variables on the level of fecal calprotectin was assessed by using a linear regression. Data analyses were performed by using a model assessing the effect of patient characteristics on the level of fecal calprotectin. The following model was used:


$$\begin{array}{l} calprotectin_{ijklm}=\beta_{0}+\beta_{1}bw+\beta_{2}age+\;\beta_{3}pweek+gender_{i}\\ \;+\;feeding_{j}+center_{k}+delivery_{l}+status_{m}\\ \;+e_{ijklm} \end{array}$$

where *calprotectin* is the level of fecal calprotectin measured for *i*th gender, *j*th feeding type, at *k*th center, *l*th delivery type and *m*th colicky status, β's are regression coefficients and *e* is random residual error. The *bw* is the patient's body weight, *age* is the age of the patient upon enrollment, *pweek* is the week of pregnancy at which the birth occured, *gender* is the gender of the patient (male, female), *feeding* is the type of feeding (breast, formula, mixed), *center* is the treatment place (1 or 2), *delivery* is the delivery type (vaginal, cesarean), and *status* is the colicky status (non-colicky, colicky). The significance of the differences between important categorical variables was assessed using estimated marginal means using the emmeans package (27) and presented averaged over the other categorical variables included in the model.

#### Infantile colic diagnosis based on fecal calprotectin level

The conditional inference tree method as implemented in packages party (28) and partykit (29) was used to determine the accuracy of diagnosing IC based on the measured fecal calprotectin level in comparison to the diagnosis based on using the Wessel criteria. For that, patients were split into two groups: calprotectin below 100 μg/g and above or equal to this value. Then the whole dataset was randomly split into training and test sets using the caret package (30). The training set included 80% of all observations. The model was then tested using the assignment to the calprotectin group to predict the colicky status. Additionally, the effect of the delivery status on prediction accuracy was evaluated. The overall prediction accuracy for both models was calculated as the mean of correctly predicted colicky status in the test set. The performance of the prediction method was assessed based on the calculating area under the receiver operating characteristic curve (ROC) curve based on the ratio of true positive to false positive predictions, accuracy (the fraction of the correct predictions) and the error rates (fraction of incorrect predictions).

## Results

### Patient characteristics and crying behavior

There were no significant differences between the group of non-colicky and that of colicky infants in terms of gender, delivery, feeding, and gestational age. Colicky infants had a slightly higher birth weight and were on average about 2 days younger at enrollment to the study (Table 1).

**Table 1:** Baseline characteristics of non-colicky and colicky infant groups.

		**Non-colicky (*n* = 95)**	**Colicky infants (*n* = 100)**	***P*-value**
Basic characteristics	Gender^1^ (female/male)	48/47	46/54	*p* ≥ 0.05
	Delivery^1^ (vaginal/cesarean)	68/38	62/27	*p* ≥ 0.05
	Feeding^1^ (breast/formula/mixed)	58/25/12	62/28/10	*p* ≥ 0.05
	Birthtime in pregnancy^2^ (week)	39.36 (SE 0.22)	39.71 (SE 0.21)	*p* ≥ 0.05
	Birthweight^2^ (g)	3,200.42 (SE 36.24)	3,443.60 (SE 32.93)	*p* < 0.01
	Age at enrollment^2^ (days)	33.14 (SE 0.50)	31.28 (SE 0.71)	*p* < 0.05
Crying behavior	Crying days^3^ last 3 weeks^2^	0.00 (SE 0.00)	14.38 (SE 0.2)	*p* < 0.01
	Avg. crying duration (min/day) last 3 weeks^2^	85.04 (SE 2.41)	219.90 (SE 2.18)	*p* < 0.01
	Avg. crying phases/day last 3 weeks^2^	4.07 (SE 0.08)	5.93 (SE 0.10)	*p* < 0.01

1 Significance of differences between count data assessed with chi-squared data.

2 Significance of differences for continous variables assessed with t-test.

3 Crying day defined as day with crying duration ≥3 h.

Crying days during the last 3 weeks, average crying duration per day and average number of crying phases per day in the group of colicky infants were all significantly elevated compared to those in the group of non-colicky babies (Table 1).

### Fecal calprotectin levels

**Figure 1:**
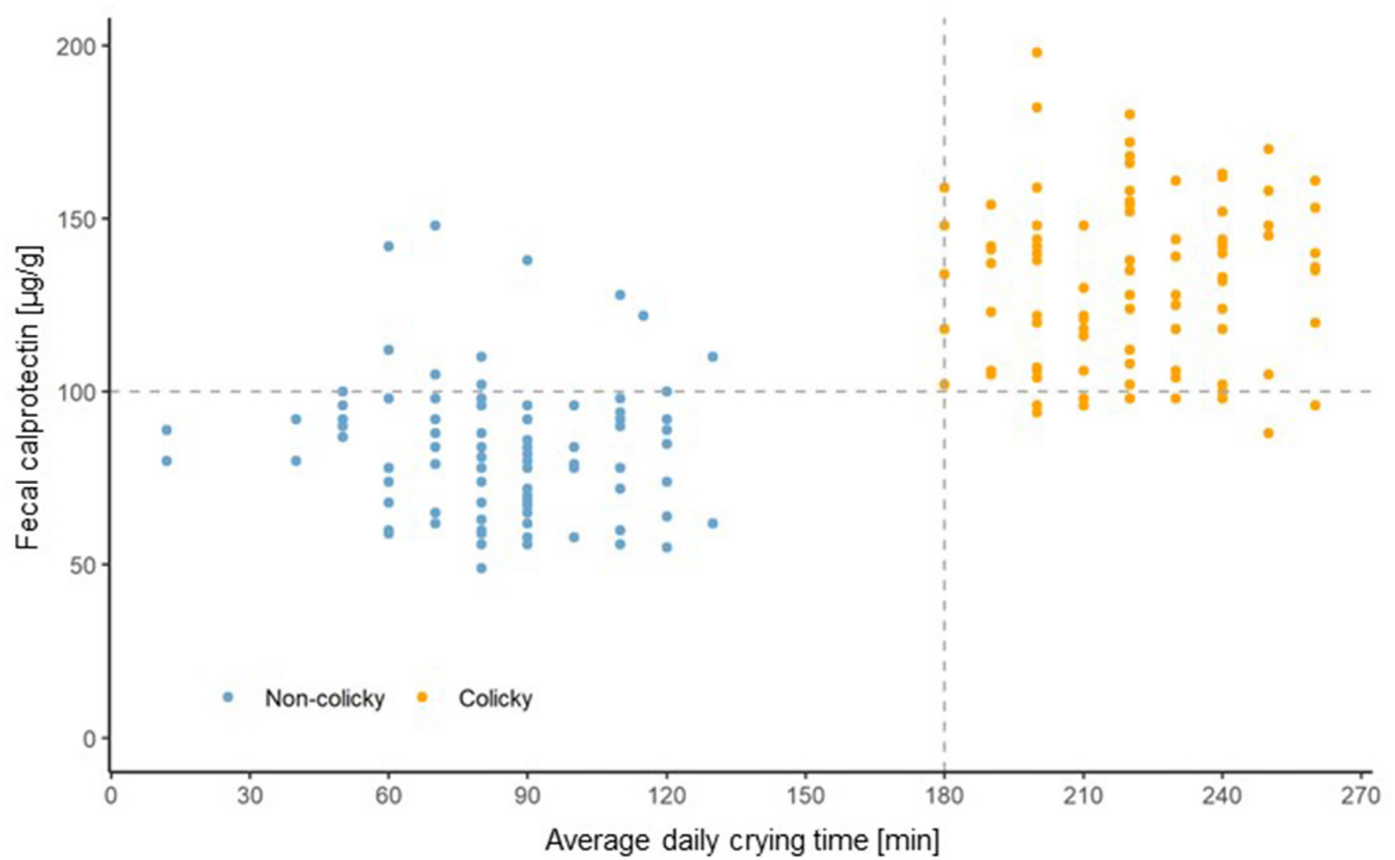
Average daily crying duration plotted vs. fecal calprotectin levels in non-colicky and colicky infants.

The average daily crying time was plotted against the fecal calprotectin levels (Figure 1). Using a fecal calprotectin level of 100 μg/g as a cut-off indicates that most of the non-colicky babies have fecal calprotectin levels below this cut-off and the majority of the colicky babies have levels above that value. It is of interest to note that 9 of the 10 non-colicky babies with fecal calprotectin levels ≥100 μg/g were delivered by cesarean section. The only infant in that group who had been born vaginally had a fecal calprotectin level of 102 μg/g, thereby making it just above the cut-off level. Statistical analysis using the model described under materials and methods revealed that the average fecal calprotectin level of colicky babies is significantly higher (46.7 μg/g; *p*-value < 0.0001) compared to that of non-colicky infants.

**Figure 2:**
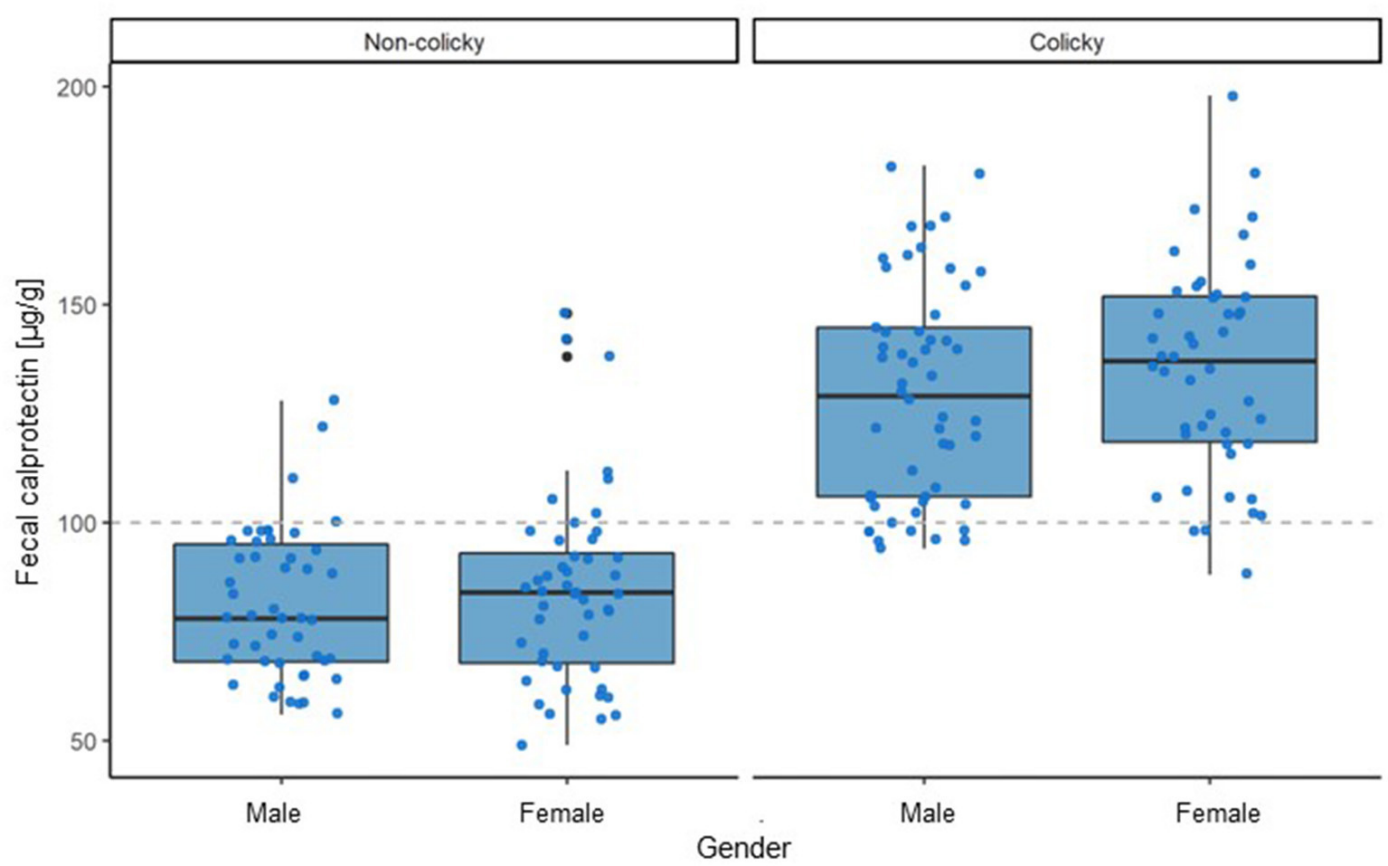
Influence of gender on fecal calprotectin levels in colicky and non-colicky infants.

Analyzing the gender dependence of the average fecal calprotectin levels in the groups of non-colicky and colicky infants revealed no significant differences within each of the two groups (Figure 2).

**Figure 3:**
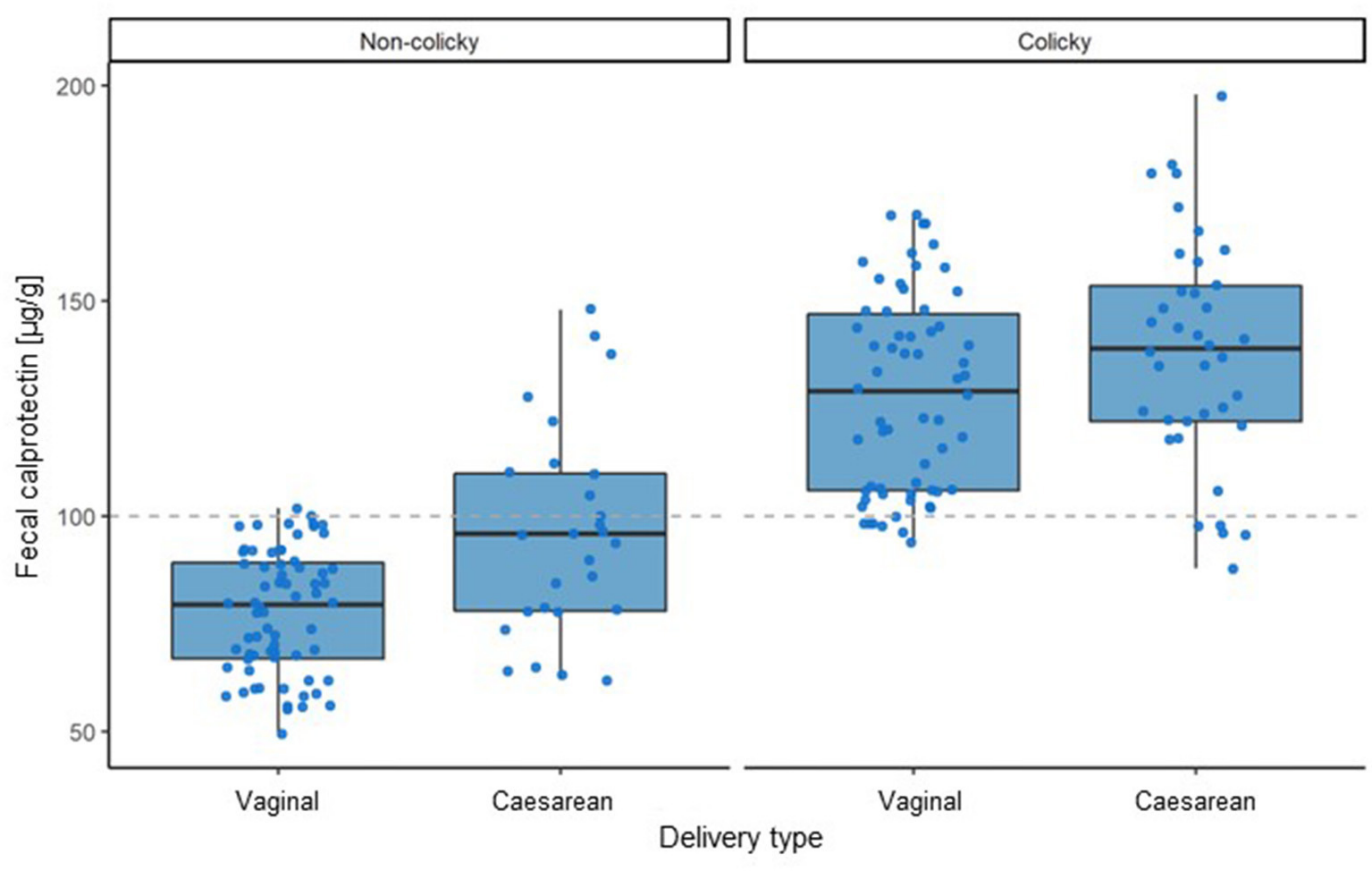
Influence of type of delivery on fecal calprotectin levels in colicky and non-colicky infants.

Analyzing the impact of the type of delivery (vaginal vs. cesarean) showed that the average fecal calprotectin level in infants born by cesarean section was elevated in non-colicky as well as in colicky babies (Figure 3). Statistical analyses revealed that this elevation was small (14.5 μg/g) but highly significant (*p*-value < 0.0001).

**Figure 4:**
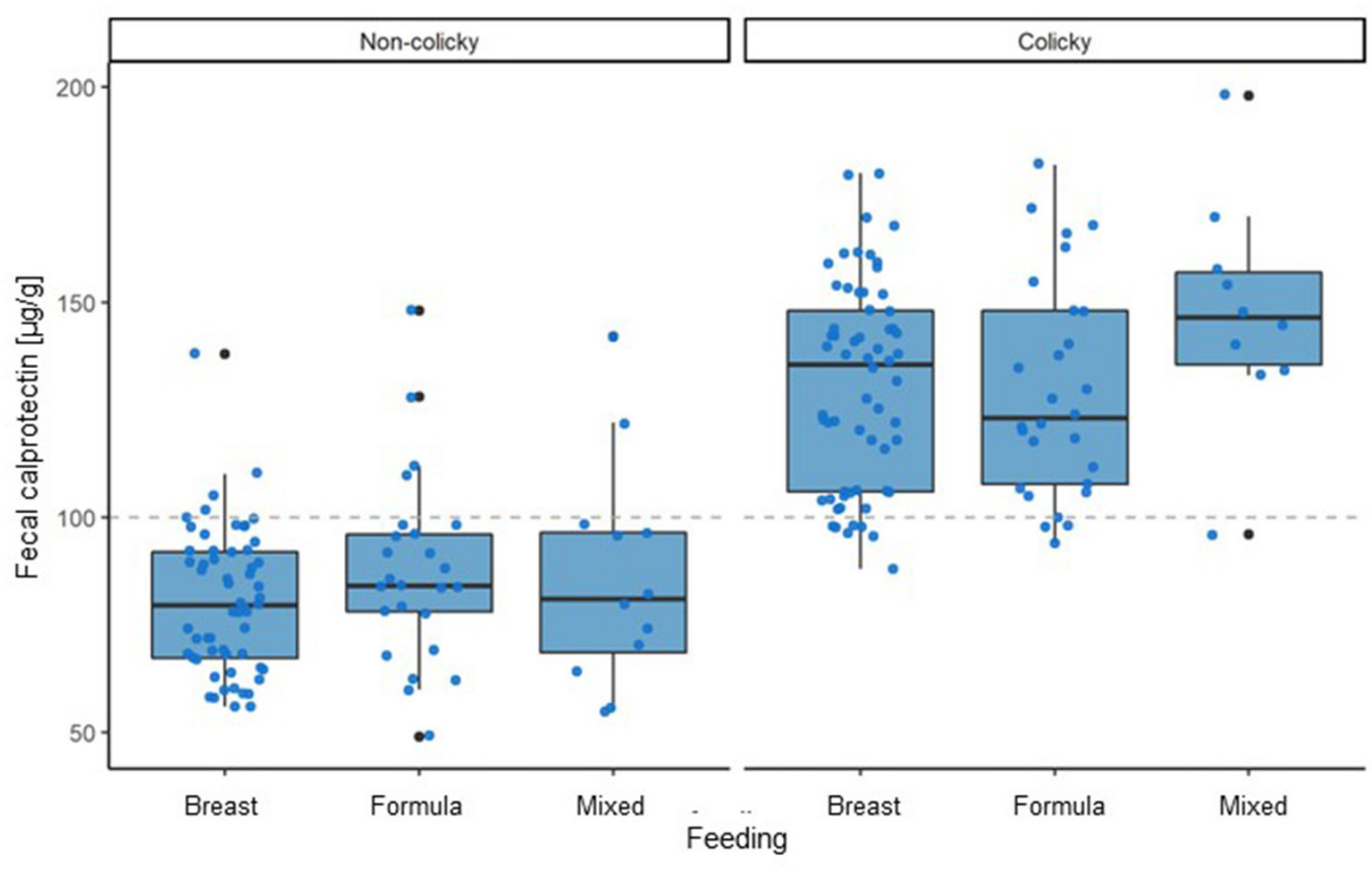
Influence of type of feeding on fecal calprotectin levels in colicky and non-colicky infants.

Analyzing the impact of the feeding type on average fecal calprotectin levels did not show any significant differences (Figure 4).

Additional statistical analyses revealed that fecal calprotectin levels were not significantly influenced by gestation age and weight at birth (data not shown).

**Figure 5:**
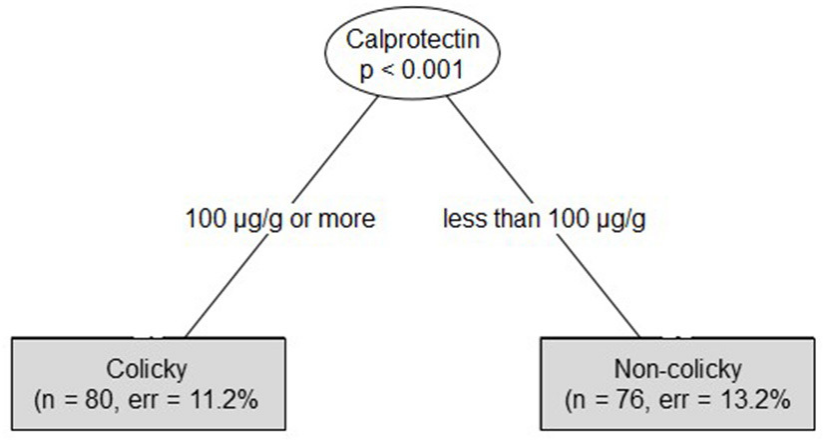
Conditional inference tree using fecal calprotectin level data to determine the colicky status of an infant.

Using the conditional inference tree method revealed that measurement of a fecal calprotectin level ≥100 μg/g shall allow to diagnose colicky status of this patient with an overall accuracy of 90%. The risks for diagnosing a wrong non-colicky status or a wrong colicky status would be 13.2 and 11.2%, respectively (Figure 5). Calculating the area under the ROC curve equaled 0.9.

**Figure 6:**
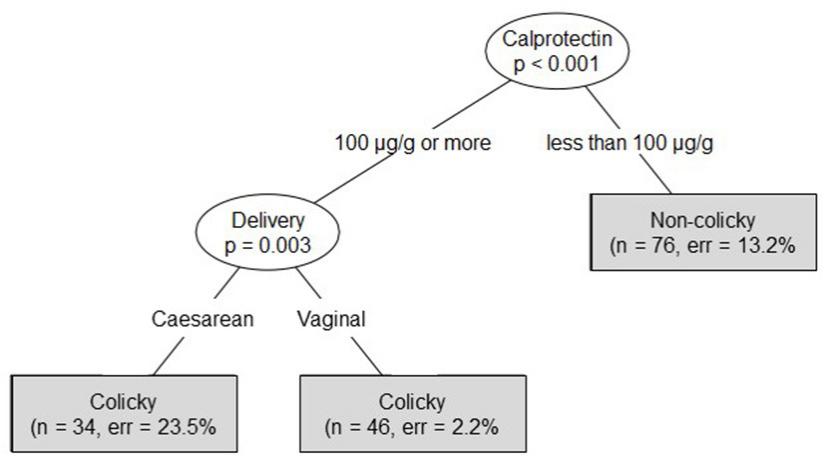
Conditional inference tree using fecal calprotectin level data and information about the type of delivery to determine the colicky status of an infant.

If the information about their type of delivery was also taken into account, it would allow a correct diagnosis of a colicky status of a vaginally born infant with 97.8% accuracy for infants with fecal calprotectin levels above 100 μg/g, while the accuracy of diagnosing the correct colicky status in a cesarean-born infant would drop to 76.5% (Figure 6). The area under the ROC curve was determined to be 0.92.

## Discussion

Reporting the crying behavior of infants by using diaries is a demanding task for the already stressed parents of these babies. It has been shown that diary reporting of crying time is reasonably accurate, while other crying parameters, e.g., the number of crying phases, is less reliable (31). Nevertheless, crying diaries are still the most commonly used tool to assess infant crying in clinical trials while the usage of recording devices has mainly been used to check on the accuracy of diary reporting (32, 33). Outside of the clinical trial setting diaries are very rarely used by practicing pediatricians, as reporting is assumed to be too demanding for parents and data analysis too demanding for physicians. In addition, reporting crying behavior for 1–3 weeks to confirm the diagnosis is also not practical in day-to-day practice. A recently published survey showed that most pediatricians are diagnosing IC based on parents' complaints about the crying of their infants without using any tools (diaries or recording devices) to support their assessment (12). The same study also found that there is variability in the diagnosis criteria employed by practicing pediatricians. Diagnostic criteria range from Wessel criteria, modified Wessel criteria, Rome III criteria, Rome IV criteria, to mixtures combining elements of these criteria.

Data from the present study demonstrate that PoC measurements of fecal calprotectin can support the diagnosis of IC. The results are also contributing to the growing evidence that the etiology of IC might be related to a gut inflammation caused by a disturbed gut microbiota. Using the PoC analysis of fecal calprotectin employed in the study can provide data allowing the support of IC diagnosis with an overall accuracy of some 90%. An interesting observation made in the study was that some non-colicky infants born by cesarean section exhibit high (above 100 μg/g) fecal calprotectin levels. Cesarean-born infants have been shown to have a dysbiosis of the gut-microbiota (34) and to have elevated fecal calprotectin levels (35). However, as the composition of the gut microbiota was not analyzed in the present study, the present study does not provide inside into the reason for elevated fecal calprotectin levels observed in cesarean-born infants. Nevertheless, the combining of data from the fecal calprotectin measurement with the knowledge about the type of delivery is allowing diagnosis of the colicky status of a vaginally-born infant with an error of only 2.2%. As there are two potential reasons for an elevated fecal calprotectin level in cesarean-born infants (cesarean section itself and IC), fecal calprotectin-based diagnosis of IC in this patient group is associated with a lower accuracy rate (76.5%).

If well organized, results of fecal calprotectin measurements can become available fast enough so as not to require an additional consultancy appointment. Taking the results from fecal calprotectin measurements into account will allow pediatricians to add to their assessment of the IC status made on the basis of information collected from parents about the crying behavior of the infant. Thereby, the pediatrician/parent interaction can benefit from the hard facts obtained by this objective measurement.

Based on the presented results it is suggested that pediatricians should consider PoC measurement of fecal calprotectin to support their diagnosis of IC. As demonstrated, fecal calprotectin levels can support the assessment of the colicky status of an infant presented by its parents. This will allow to move the diagnosis from extracting information from stressed parents toward a more objective clinical measurement. While the PoC measurement of fecal calprotectin is relatively easy and fast, there remains the problem that this measurement might not be reimbursed by the health insurance for the baby, at least in most of the cases. However, based on our experience, a lot of parents with colicky babies are very thankful for being provided with data from a clinical measurement indicating that the colicky status of their beloved baby is most likely not caused by their parental behavior.

## Data availability statement

Datasets analyzed for this study can be found in FigShare at: https://figshare.com/articles/dataset/Supporting_the_Diagnosis_of_Infantile_Colic_by_a_Point_of_Care_Measurement_of_Fecal_Calprotectin/20132231.

## Ethics statement

The studies involving human participants were reviewed and approved by Bioethics Committee at the University of Kalisz. Written informed consent to participate in this study was provided by the participants' legal guardian/next of kin. Written informed consent was obtained from the minor(s)' legal guardian/next of kin for the publication of any potentially identifiable images or data included in this article.

## Author contributions

MB, MP, HS, JP, and HK: conceptualization. MB, MP, and HK: methodology. MP: software. JP and MP: validation. MP and HK: formal analysis. MB and HK: investigation. JP: resources. MB, JP, and MP: data curation. HS and MP: writing—original draft preparation. JP, MP, and HS: writing—review and editing. MP and HS: visualization. JP: supervision. JP: project administration. All authors contributed to the article and approved the submitted version.

## Conflict of interest

All authors declare that the research was conducted in the absence of any commercial or financial relationships that could be construed as a potential conflict of interest.

## Publisher's note

All claims expressed in this article are solely those of the authors and do not necessarily represent those of their affiliated organizations, or those of the publisher, the editors and the reviewers. Any product that may be evaluated in this article, or claim that may be made by its manufacturer, is not guaranteed or endorsed by the publisher.
